# Scale‐dependent influences of environmental, historical, and spatial processes on taxonomic and functional beta diversity of Japanese bat assemblages

**DOI:** 10.1002/ece3.11277

**Published:** 2024-04-15

**Authors:** Takahiro Maki, Nozomi Sannomiya, Toshihide Hirao, Dai Fukui

**Affiliations:** ^1^ Amami Station, International Center for Island Studies Kagoshima University Kagoshima Japan; ^2^ The University of Tokyo Forests, Graduate School of Agricultural and Life Sciences The University of Tokyo Tokyo Japan; ^3^ The University of Tokyo Chichibu Forest, Graduate School of Agricultural and Life Sciences The University of Tokyo Saitama Japan; ^4^ Fuji Iyashinomori Woodland Study Center, Graduate School of Agricultural and Life Sciences The University of Tokyo Yamanashi Japan

**Keywords:** bat assemblage, beta diversity, biogeographical borders, community assembly, Japanese archipelago

## Abstract

This study investigated the relative influences of environmental, spatial, and historical factors, including the island‐specific history of land connectivity, on bat assemblages in the Japanese Archipelago. We collected bat distribution data from 1408 studies and assigned them to Japan's First Standard Grid (approximately 6400 km^2^). Japanese bat assemblages were analyzed at two scales: the entire Japanese Archipelago comprised 16 islands and exclusively the four main islands. At first, we calculated taxonomic and functional total beta diversity (*β*_total_) by Jaccard pairwise dissimilarity and then divided this into turnover (*β*_repl_) and richness‐difference (*β*_rich_) components. We conducted hierarchical clustering of taxonomic beta diversity to examine the influence of the two representative sea straits, Tsugaru and Tokara, which are considered biogeographical borders. Variation partitioning was conducted to evaluate the relative effects of the three factors on the beta diversity. Clustering revealed that the Tokara Strait bordered the two major clades; however, the Tsugaru Strait did not act as a biogeographical border for bats. In the variation partitioning, shared fraction between spatial and historical factors significantly explained taxonomic and functional *β*_total_ and taxonomic *β*_repl_ at the entire archipelago scale, but not at the four main islands scale extending only Tsugaru Strait but not Tokara Strait. Pure environmental factors significantly explained functional *β*_total_ at both scales and taxonomic *β*_total_ only at the four main islands scale. These results suggest that spatial and historical factors are more pronounced in biogeographical borders, primarily structuring assemblage composition at the entire archipelago scale, especially in taxonomic dimension. However, current environmental factors primarily shape the assemblage composition of Japanese bats at the main island scale. The difference in results between the two scales highlights that the primary processes governing assemblages of both dimensions depend on the quality of the dispersal barriers between terrestrial and aquatic barriers for bats.

## Graphical abstract

This study investigated the relative influences of environmental, spatial, and historical factors, including the island‐specific history of land connectivity, on bat assemblages in the Japanese Archipelago. Spatial and historical factors are more pronounced in biogeographical borders, primarily structuring assemblage composition of Japanese bats at the entire archipelago scale, especially in taxonomic dimension. However, current environmental factors primarily shape the assemblage composition at the main four islands scale. The difference in results between the two scales highlights that the primary processes governing assemblages of both dimensions depend on the quality of the dispersal barriers between terrestrial and aquatic barriers for bats.

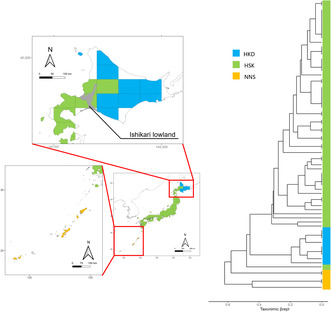

## 1 INTRODUCTION

Understanding the process that shapes biodiversity is a fundamental concern in community ecology and biogeography (Lomolino et al., 2017; Mittelbach & McGill, 2019). At a macro‐scale, spanning from landscapes to continents, three key processes—niche‐based, spatial, and historical—drive the structural pattern of biodiversity. Niche‐based processes function on the premise that individual species adapt to specific environmental conditions (Leibold et al., 2004). These processes deterministically drive biological communities alongside environmental factors such as climate and geographical features (Di Febbraro et al., 2022; Ding et al., 2023). Spatial processes shape communities via dispersal limitations and stochastic ecological drift (Dray et al., 2006; Hubbell, 2001). Historical processes shape present‐day community patterns through historical legacies, such as paleoclimate (Svenning et al., 2015). For instance, assemblages of New World birds, mammals, and amphibians exhibit stronger nesting patterns in high‐latitude areas, which are younger due to deglaciation, than in low‐latitude areas due to past extinctions and recent recolonization (Dobrovolski et al., 2012). In summary, niche‐based, spatial, and historical processes correspond to environmental, spatial, and historical factors, respectively (Buonincontri et al., 2023; García‐ Navas et al., 2023).

Beta diversity serves as a valuable indicator of biodiversity patterns, revealing the influences of these factors. Beta diversity, initially defined by Whittaker (1961) as “the extent of change in community composition, or degree of community differentiation, in relation to a complex gradient of environment, or a pattern of environments,” has been widely employed as a metric to elucidate the spatial and temporal dynamics of biodiversity in numerous studies (e.g., Dobrovolski et al., 2012; Varzinczak et al., 2018). This measure enables us to discern patterns of species composition changes in relation to specific factors and explore their interrelationships. Beta diversity in a functional dimension provides robust information about the effects of eco‐evolutionary processes on community assembly. Different from taxonomic diversity, which is measured in units of nomenclatural species, functional diversity is measured to explore the range and traits representing the functional strategy of each species within a community (Mittelbach & McGill, 2019). Thus, the functional beta diversity can clarify the dissimilarity in functional strategies among communities (Villéger et al., 2013). Partitioning beta diversity into its components of replacement (turnover) and richness difference (nestedness) is a method that provides a deeper understanding of the mechanisms driving community assembly (Cardoso et al., 2014; Monadjem et al., 2023). The replacement component represents turnover of the community composition in terms of both species and functional traits along an ecological gradient, depending on the niche breadth of each species (Legendre, 2014). The richness‐difference component reflects the differences in diversity of niche availability among communities according to ecological processes (Legendre, 2014).

Spatial, environmental, and historical factors exert partially independent effects on beta diversity, they also share influence among or between one another. Employing statistical methods that simultaneously examine the relative influences of specific variables on variations in community composition, such as beta diversity, allows us to infer the relative significance of individual and shared effects of each factor separately (Peres‐Neto et al., 2006). Our understanding of the relative influence of these three factors on community dynamics is growing (e.g., Gavilanez & Stevens, 2013; Rezende et al., 2018; Varzinczak et al., 2018).

In island communities characterized by substantially higher endemism than continental regions, the history of terrestrial connectivity and isolation emerges as a significant driver of community structure (Lomolino et al., 2017; Nakamura et al., 2009). In certain regions, the influence of this factor is recognized as a biogeographical boundary, as seen with the Wallace Line (Lohman et al., 2011). Consequently, the composition of island communities may be shaped by complex interactions among these processes, including the island‐specific historical factor. However, our understanding of the relative influence of these three factors in island ecosystems remains limited (Kubota et al., 2014; Nakamura et al., 2009).

Bats constitute a prominent mammalian presence within global island ecosystems. Due to their remarkable ability to disperse through sea barriers, approximately 60% of bat species are found on islands, and 25% of species are insular endemics (Conenna et al., 2017; Jones et al., 2009). Like that of many other organisms, the richness of bat species has been observed to correlate with island area (Frick et al., 2008; Ricklefs & Lovette, 1999). The composition of bat species in island ecosystems is influenced by several key factors, including the distance between islands (Presley & Willig, 2008), the level of isolation, and the island area (Lavery et al., 2016). Due to their extensive dispersal abilities through flight, bat diversity patterns are predominantly shaped by niche‐based processes, setting them apart from other small mammals in continental regions (Bae Heidrich et al., 2020; Bosso et al., 2016; Monadjem et al., 2023; Varzinczak et al., 2019). However, a genetic study of island bats in the Philippines has revealed that coastlines during the Late Pleistocene period had a more pronounced impact on genetic distance among bat populations than the present shoreline (Heaney et al., 2005). This intriguing finding underscores the influence of inter‐island distances (Presley & Willig, 2008) and past shorelines (Heaney et al., 2005) on bat populations. It suggests that despite their ability to fly, sea barriers can still hinder bat dispersal, and historical and geological processes related to past terrestrial connections and isolation play a defining role in shaping bat diversity patterns. Consequently, exploring the relative significance of historical factors, including the history of land connections among islands and environmental and spatial factors, provides valuable insights into the pivotal role of historical processes in shaping the diversity patterns of bats in island ecosystems.

**FIGURE 1:**
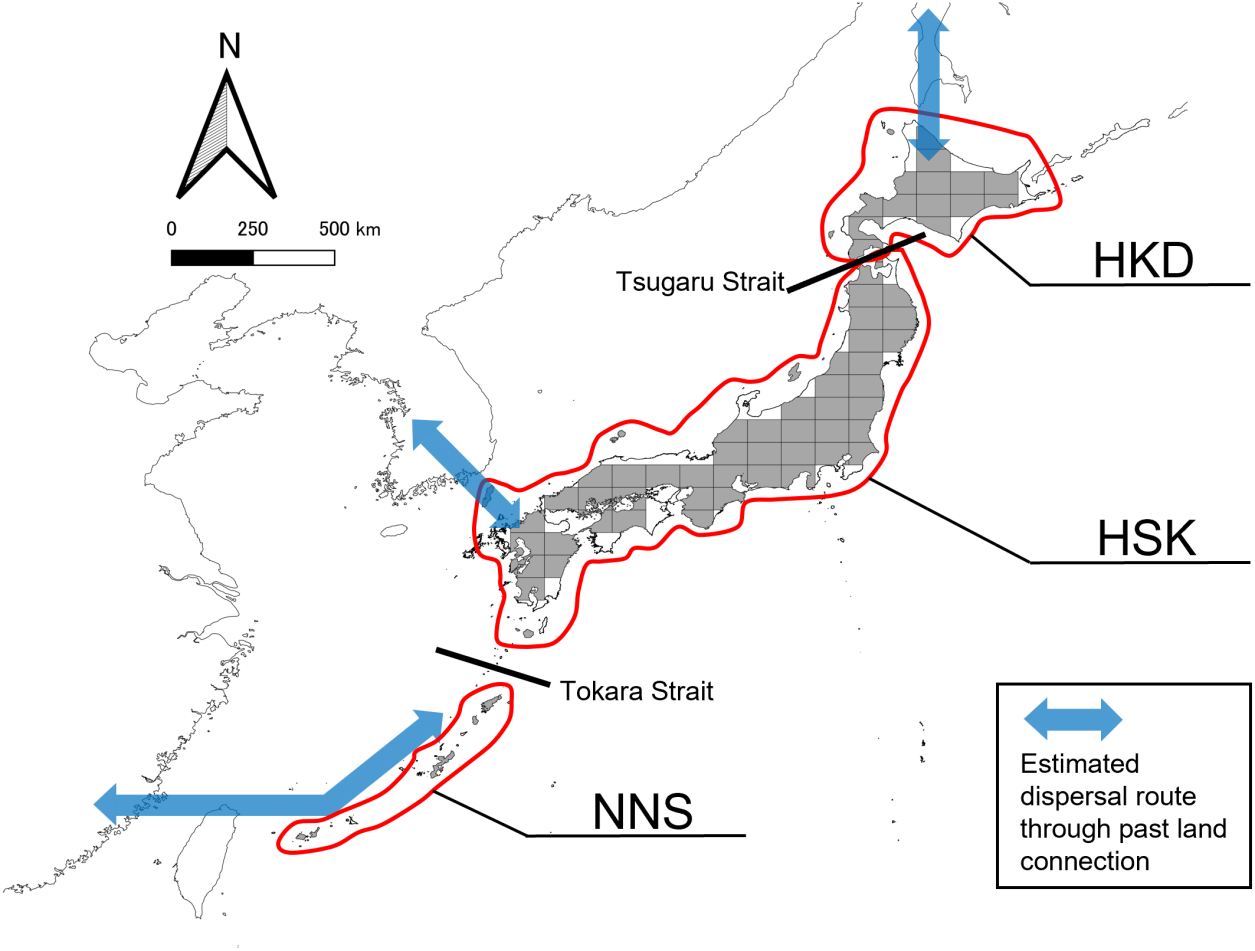
Map of the regions in the archipelago in this study. Solid black lines indicate the primary biogeographical borders, Tsugaru Strait and Tokara Strait, dividing three regions: Hokkaido (HKD), Honshu‐Shikoku‐Kyushu (HSK), and Nansei Islands (NNS). Gray locations indicate examined assemblages including 59 grids covering 6400 km^2^ and 12 islands. Blue arrows indicate the estimated dispersal route through past land connections (Kaito & Toda, 2016; Millien‐Parra & Jaeger, 1999).

The Japanese Archipelago is suitable for examining the relative contributions of these factors, including island‐specific factors, for several reasons. Most of the islands in this archipelago are land‐bridge islands that have historical connectivity with the Eurasian continent (Osozawa et al., 2012). According to previous studies (Kawamura, 2007; Motokawa & Kajihara, 2017), these islands can be categorized into three main regions: Hokkaido (HKD), Honshu‐Shikoku‐Kyushu (HSK), and the Nansei Islands (NNS; Figure 1). HKD is a major northern island of the Japanese Archipelago and is separated from Honshu Island, which belongs to HSK, by the Tsugaru Strait. This strait has been recognized as a biogeographical border named “Blakiston's line.” HSK has been separated from the East Asian continent (Korean Peninsula) since the Miocene, whereas HKD has been separated more recently; the northern part of Hokkaido Island has frequently been connected to the continent after the Miocene. NNS is separated from HSK by the Tokara Strait, which is recognized as a biogeographical border called the “Watase line.” NNS is connected to the southern Asian continent and has been separated from the continent for longer time than the other regions (Motokawa & Kajihara, 2017; Osozawa et al., 2012). Consequently, these islands exhibit a higher degree of endemism. The variation in geological history among these regions influences species composition through continental species dispersal and speciation. In the context of bats, Dobson (1994), who summarized the distribution pattern of terrestrial mammals in the Japanese Archipelago, explained that Blakiston's line does not serve as a biogeographical barrier for bats due to their flying capabilities, although it does act as a barrier for non‐flying mammals. There is a notable absence of studies exploring the impact of the Watase line on bats at the community level, and a comprehensive understanding of the relative influence of these three factors on bat diversity in the Japanese Archipelago remains limited.

In this study, our primary focus was to investigate the driving factors behind bat assemblages in the Japanese Archipelago, employing a statistical approach, variation partitioning, capable of disentangling independent and shared influences among these factors. Recognizing that the significance of sea straits as biogeographical borders can vary by taxonomic group (Kubota et al., 2014; Millien‐Parra & Jaeger, 1999; Miyamoto et al., 2018), we conducted cluster analyses on taxonomic beta diversity to reveal the influence of two representative straits, namely the Tsugaru and Tokara Straits before delving into an examination of these three key factors. To gain insights into island‐specific influences, we assessed the functional and taxonomic beta diversity of bat assemblages at two different scales: the entire Japanese Archipelago and exclusively the four main islands covering an area exceeding 18,800 km^2^, excluding the Nansei Islands separated from the main islands by the Tokara Strait. We formulated two hypotheses to guide our investigation: (1) Based on Dobson's (1994) findings, we hypothesized that the Tsugaru Strait (known as Blakiston's line) does not function as a biogeographical barrier for bat assemblages. Conversely, considering the high endemism of bats in the NNS region (Ohdachi et al., 2015; Preble et al., 2021), we posited that the Tokara Strait (referred to as the Watase line) does indeed act as a biogeographical border. Thus, we expect that in the hierarchical clustering, the first node of the dendrogram will be located in the Tokara strait and that there are no representative nodes in the Tsugaru strait. (2) Based on the above hypothesis, we anticipated that niche‐based processes would predominantly shape bat assemblages on the four main islands, given their high dispersal abilities. However, we put forward that assemblages at the broader archipelago scale would exhibit a stronger association with historical processes, reflecting past land connections, compared with those at the main island scale, primarily due to the influence of Nansei Islands. Thus, we predict that historical factors will have higher explanatory power on taxonomic diversity, especially on the replacement component, at the entire‐archipelago scale than at the main islands scale, which is predicted to be explained by environmental factors as a result of variation partitioning. (3) Functional diversity reflects the composition of functional strategies and niche positions within a community, which strongly correlate with environmental gradients (Carvalho et al., 2023; Jarzyna et al., 2021; Swenson. et al., 2012) through niche‐based processes. Therefore, we predicted that environmental factor will have higher explanatory power on functional beta diversity, including replacement and richness differences, than on taxonomic diversity. In addition, we expect that historical factors will have lower explanatory power on functional diversity than on taxonomic diversity because the selective pressures of the environment on functional traits are likely to more strongly determine the functional composition than historical process such as different species sources.

## 2 MATERIALS AND METHODS

### 2.1 Study area

The Japanese Archipelago is situated east of the Eurasian continent. Several biogeographical boundaries are associated with the historical connections between the continent and surrounding islands within this archipelago. Our study encompassed a comprehensive investigation of the entire archipelago, from its north on Hokkaido Island to its south on Iriomote Island, as depicted in Figure S1. Herein, the biogeographical border of the Watase line, which has been described to exist between Kodakara Island and Akuseki Island (Yakushima Island and Amami Island in this study; Figure S1). Komaki (2021) indicated that the position of the border is ambiguous and proposed the question of whether a clear line exists. Since our primary objective was to investigate the impact of historical factors on land connections rather than to define a clear border, we opted to assume a boundary between Yakushima and the Amami Islands (Figure S1).

### 2.2 Species distribution data

We collected literature on bats in the Japanese Archipelago by the following processes. First, we collected literature referenced in Ohdachi et al. (2015) which reviewed basic information on all mammals, including distribution data in Japan. Second, we searched in Google Scholar (https://scholar.google.co.jp/schhp?hl=ja) using a combination of the following keywords: bat, bats, Chiroptera, Japan, Japanese archipelago, コウモリ. Most literature were found by the above two steps; however, some literature, such as newspapers or local papers, were provided by the researcher community. Finally, we collected more than 2000 papers on bats in the Japanese Archipelago. Of these, we selected papers that contained distribution data recorded between 1950 and 2021 with resolution higher than Japan's First Standard Grid (FSD), a national grid system covering approximately 6400 km^2^ (1° Longitude, 40′ Latitude). We used this grid system to cover bat home range size, which move more than 10 km in single night in several species (Fujioka et al., 2019; Niga et al., 2023). After these processes, distribution data were extracted from the selected 1408 papers and assigned to FSDs as presence/absence data. For our statistical analysis, we employed grids where the terrestrial area constituted more than half of each grid. In addition, we included islands where bat fauna had been thoroughly studied, as assemblages from minor islands offer valuable insights into the factors influencing community composition in island regions. Consequently, we scrutinized the bat assemblages across selected 59 grids from 136 grids where distribution data were assigned and 12 islands (Figures S1 and S2). The Japanese Archipelago hosts 37 identified species of bats (Funakoshi et al., 2022; Kobayashi et al., 2022; Ohdachi et al., 2015), except for two species that are considered to be extinct (*Pteropus loochoensis* and *Pipistrellus sturdeei*). *Murina tenebrosa* and *Taphozous melanopogon* were excluded from this study due to the limited presence of only one individual reported within the Japanese Archipelago. *Hypsugo pulveratus* was also excluded as its distribution in the Japanese Archipelago was documented in 2022 (Funakoshi et al., 2022). *Pteropus pselaphon* was not included because its distribution area, Bonin Islands, was not included in this study. Consequently, our analysis focused on 33 species of bats inhabiting the Japanese Archipelago, as detailed in Table S1. Because a clear shift in distribution has not been indicated among the Japanese bats during the period, we assumed that no range shift has occurred.

### 2.3 Functional data

Bat wing morphology is closely related to foraging habitats and strategies (Aldridge & Rautenbach, 1987; Norberg & Rayner, 1987). To calculate functional beta diversity, we used four wing variables: forearm length, aspect ratio, relative wing loading, and wing tip shape index. These variables were measured from museum and private specimens. Sample sizes ranged from 1 to 79 per species. Forearm length is used as a proxy for body size (Penone et al., 2018; Thiagavel, 2017; Wu et al., 2014). Aspect ratio represents energy efficiency of flight (Norberg & Rayner, 1987), and related traits such as habitat selection (Aldridge & Rautenbach, 1987; Roemar et al., 2019). High value of wing loading indicates high flight speed (Norberg & Rayner, 1987). We used relative wing loading, which enabled us to measure the wing loading corrected for body mass to obtain variables independent of body mass (Norberg et al., 2000). Wing tip shape index describes the roundness and sharpness of wing shape and represents flight maneuverability (Findley et al., 1972; Norberg & Rayner, 1987).

### 2.4 Beta diversity

Beta diversity and its components were calculated based on the framework of Podani and Schmera (2011), Carvalho et al. (2012), and Cardoso et al. (2014) with the Jaccard index. We calculated the taxonomic and functional pairwise beta diversity between each of the assemblages (FSDs). The taxonomic and functional beta diversity (*β*_total_) was divided into replacement (*β*_repl_) and richness‐difference (*β*_rich_) components. To calculate these variables, we used the beta function of the R package “BAT” (Cardoso et al., 2015). Functional beta diversity was calculated using a functional dendrogram (tree) based on the Unweighted Pair Group Method with Arithmetic Mean (UPGMA). To obtain a dissimilarity matrix of functional traits for calculation of functional diversity, we used gawdis‐based functional distance by function *gawdis*, using the package “gawdis” (de Bello et al., 2021). This functional distance provides optimized weights that minimize differences in the correlation among traits (Carvalho et al., 2023; de Bello et al., 2021), and this distance was used to describe the functional tree.

### 2.5 Determining biogeographical border

To examine the two sea straits Tsugaru and Tokara, which act as biogeographical borders, we clustered community compositions based on their dissimilarity. Based on an instructive study that indicated a methodological roadmap of biogeographical regionalization (Kreft & Jetz, 2010), we used hierarchical clustering analysis with the UPGMA algorithm. We used the taxonomic *β*_repl_ due to its suitability for biogeographical regionalization, particularly in tasks like determining biogeographical borders, where it is essential to account for richness‐independent species turnover (Cardoso et al., 2014; Kreft & Jetz, 2010). To visualize the influence of the biogeographical borders, we colored the clusters that branched from first node between NNS and HSK and between HSK and HKD. We also conducted clustering on taxonomic *β*_total_ and *β*_rich_ to compensate for the multiple perspectives of beta diversity. Species‐based clustering using presence/absence data of assemblages measured by Euclidean distance was also conducted to determine distribution patterns.

### 2.6 Environmental, spatial, and historical factors

To examine the influence of the present environment, the mean annual temperature, annual precipitation, mean elevation, mean slope, and island area were used as covariates. In addition to these climatic and topological variables, anthropogenic factors such as urbanization and land‐use conversion have been considered crucial for shaping the community composition of bats (Cisneros et al., 2016; Mena et al., 2022). Thus, the ratio of human residential areas, including urban and agriculture, to land area and plantations to forest area was added as environmental factors. The climate data were extracted from the 1‐km gridded data in the Mesh climate data 2010 (JMA, 2012), and land‐use data were obtained from the national land‐use/cover database (Ogawa et al., 2013). We obtained mean variables of each factor within each FSD as explanatory variables by calculation of 1‐km grids within each of FSDs by *summarize* function in “dplyr” package (Wickham et al., 2023).

Moran's eigenvector maps (MEMs) were used as spatial factors (Dray et al., 2006, 2021; Monadjem et al., 2023). This method describes the spatial factors among sites at a multiscale level. First, the centroids of each grid and island are determined. A spatial weight matrix (SWM) representing the network connections among the study grids was created based on the distance between the centroids. This study tested four configurations as representations of network linking: Delaunay triangulation, Gabriel, relative neighborhood, and minimum spanning tree (Borcard et al., 2018). The SWM with the highest adjusted R^2^ value was selected among the four methods. The best SWM was used to calculate statistically significant spatial eigenvalues (MEMs) using the function *listw.select* from the package “adespatial” (Dray et al., 2021).

Historical factors, such as past climate and land connections, have also been considered crucial for mammalian community composition (Fukasawa & Akasaka, 2019; Rowan et al., 2016; Srinivasan et al., 2014). Mammals in the Japanese Archipelago were potentially impacted by two climatic events, Bølling‐Allerød interstadial and Younger Dryas Stadial. In the Bølling‐Allerød interstadial at approximately 14,700–12,900 year Before Present (BP), the climate became warmer (Fordham et al., 2017; Shen et al., 2010), and the climate shift impacted mammal composition (Berto et al., 2019). Japanese mammals also experienced extinctions around that time (Kawamura, 2007). In the Younger Dryas Stadial, the temperature suddenly dropped at approximately 12,860–11,640 year BP, and the climate became dry, causing vegetation to change (Nakagawa et al., 2005). This study used the mean annual temperature and precipitation during these two periods as past climatic factors. The differences between each two climatic variables between the Bølling‐Allerød interstadial and the Younger Dryas Stadial, and between the Younger Dryas Stadial and the current climate were compiled as historical variables in the following statistical analysis to avoid multicollinearity among these climatic variables. Thus, four climatic variables were used as historical factors in this study. These past climate data were obtained from Brown et al. (2018) in 2.5 min, based on Fordham et al. (2017). We obtained mean variables of each factor within each FSD as explanatory variables by calculation of 2.5‐min grids within each FSD using the *extract* function in the “raster” package (Hijmans, 2023). We integrated information about the Tsugaru and Tokara straits concerning the history of land connections into our analysis. To achieve this, we categorized these variables into Tsugaru and Tokara. For the Tsugaru variable, we assigned a value of 1 to grids and islands north of the Tsugaru Strait, while the rest were assigned a value of 0. Similarly, islands located south of the Tokara Strait were assigned a value of 1 for the Tokara variable, whereas all others were assigned a value of 0.

### 2.7 Examining the relative contribution of three factors

The influences of these three factors on patterns of bat diversity were examined at two scales: the entire Japanese Archipelago and only the four main islands larger than 18,800 km^2^, excluding the NNS region. We compared the outcomes of these analyses. To ascertain which of the three factors primarily influenced the present composition of bat assemblages, we employed variation partitioning analysis through distance‐based redundancy analysis (dbRDA). This method allows us to determine the relative contributions of these factors to the variation (dissimilarity) in assemblage composition. Taxonomic and functional *β*_total_, *β*_repl_, and *β*_rich_ were used as response variables. After partitioning the independent contributions of the three factors, we assessed their statistical significance using the null model approach. Specifically, we conducted 9999 permutations of the rows in the original bat assemblage matrix, performing a variation partitioning procedure for each iteration to obtain new adjusted *R* ^2^ values. Subsequently, we examined the probability that the original *R* ^2^ values were greater than the *R* ^2^ values obtained by chance, adopting a significance level of 0.05 (Tello & Stevens, 2010; Varzinczak et al., 2018). For all statistical analyses, we utilized the R 4.0.3 environment for statistical computing (R Core Team, 2022), along with its extended packages vegan (Oksanen et al., 2022) and ade4 (Dray & Dufour, 2007).

## 3 RESULTS

### 3.1 Biogeographical borders

**FIGURE 2:**
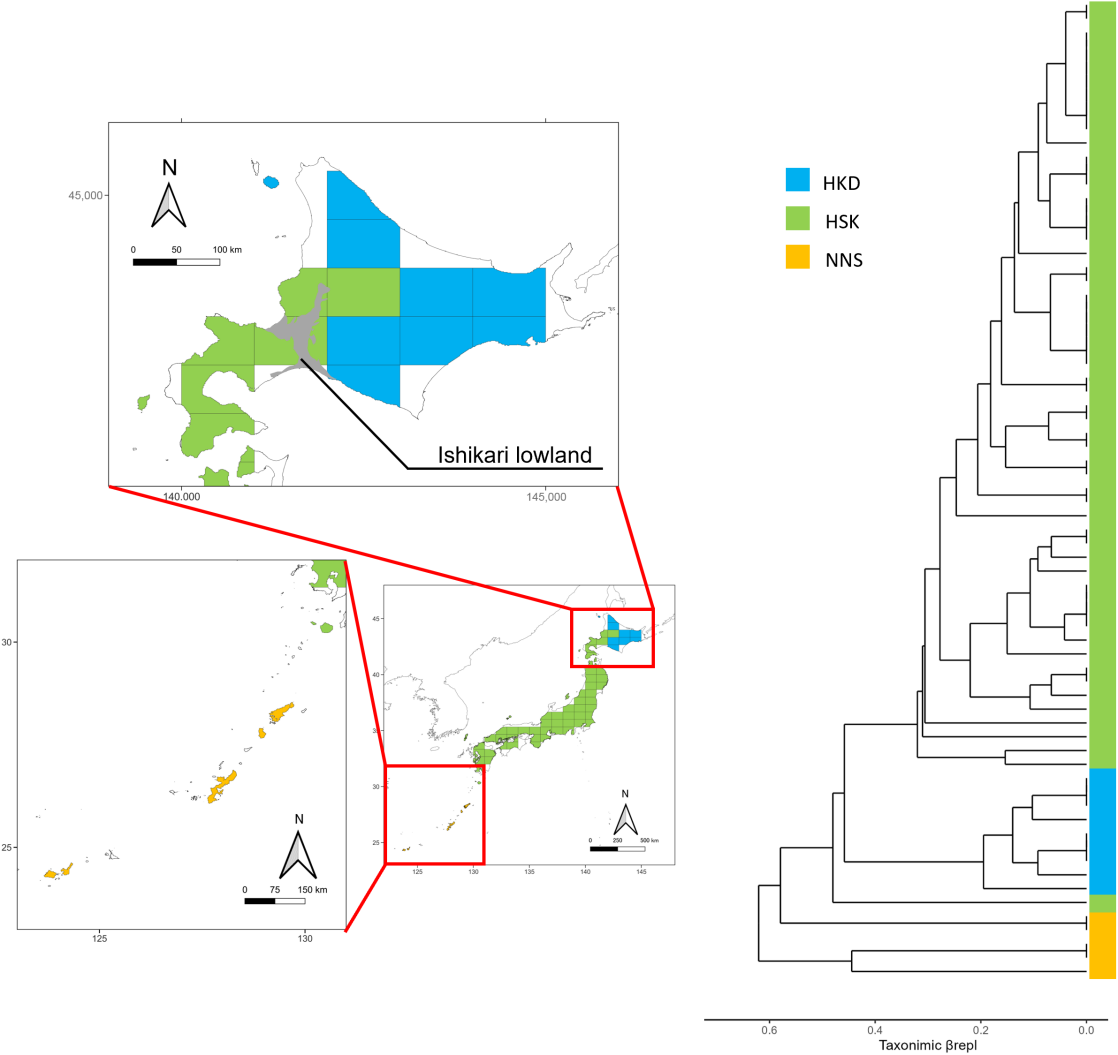
Results of cluster analysis based on taxonomic turnover (*β*_repl_) in the dendrogram and map of the Japanese Archipelago. The assemblages are colored according to the clades determined by cluster analysis. Blue is the cluster in the Hokkaido region (HKD), green is that in the Honshu‐Shikoku‐Kyushu region (HSK) and partly HKD region, and orange is that in the Nansei Islands region (NNS). The gray zone in the map indicates Ishikari lowland, which is considered to be the border dividing the green and blue clusters.

The literature survey showed that mean species richness was 10.42 ± 3.56 in each grid and island. The findings from hierarchical clustering analysis utilizing the UPGMA algorithm on *β*_repl_ revealed that the Tokara Strait (HKD‐HSK vs. NNS; Figure 2, Figure S3) bordered the two major clades. Among the clusters in HKD and HSK, Kuchinoerabu island branched first from others. A second node was identified in central‐western Hokkaido, visualized in Figure 2 with different colors. The Tsugaru Strait did not serve as a borderline between these major clades. The result of clustering on *β*_rich_ did not show the borders among the three regions and reflected species richness differences (Figure S4). The results of clustering in *β*_total_ were mostly consistent with those in *β*_repl_; however, some assemblages were posited in other regions (Figure S5). The results of species‐based clustering showed that the species inhabiting only HKD were clearly clustered, and the species in only NNS were clustered with three species, *Pteropus dasymallus*, *Myotis rufoniger*, and *Tadarida latouchei* (Figure S6).

### 3.2 Factors driving the assemblages

The replacement and richness‐difference components had similar contributions to both taxonomic diversity (*β*_repl_ = 0.32 ± 0.23, *β*_rich_ = 0.27 ± 0.19 at the entire‐archipelago scale; *β*_repl_ = 0.27 ± 0.17, *β*_rich_ = 0.24 ± 0.17 at the main islands scale; Figure S7) and functional diversity (*β*_repl_ = 0.23 ± 0.18, *β*_rich_ = 0.20 ± 0.15 at the entire‐archipelago scale; *β*_repl_ = 0.19 ± 0.15, *β*_rich_ = 0.18 ± 0.13 at the main islands scale; Figure S7).

The shared fraction encompassing all three factors contributed significantly to the *β*_total_ of both taxonomic and functional diversity, comprising 24.5 and 23.9% of the total variation at the entire‐archipelago scale and 30.2 and 28.4% at the main islands scale, respectively. *β*_total_ for both taxonomic and functional diversity was significantly explained by purely spatial factors (11.0 and 8.4%) and shared fraction between the spatial and historical factors (10.0 and 5.9%) at the entire‐archipelago scale. The *β*_total_ for functional diversity was significantly explained by purely environmental factors (7.3%) and shared fraction between the spatial and environmental factors (4.5%). At the main islands scale, purely environmental factors significantly explained *β*_total_ for both taxonomic and functional diversity (7.4 and 8.5%).

In terms of *β*_repl_, the shared fraction encompassing all three factors contributed significantly to both taxonomic and functional beta diversity, comprising 31.1 and 32.0% of the total variation at the entire‐archipelago scale and 53.9 and 52.3% at the main islands scale, respectively. *β*_repl_ for taxonomic diversity at the entire‐archipelago scale was significantly explained by spatial and historical factors (14.6%). Other fractions of factors were not significantly explained by the three factors.

As for *β*_rich_, the shared fraction between spatial and environmental factors significantly explained taxonomic (16.2%) and functional (9.6%) beta diversity at the entire‐archipelago scale. At the main islands scale, *β*_rich_ was explained by the three factors less than the other index of beta diversity and scale (Table 1).

**TABLE 1:** Influence of each factor on taxonomic and functional beta diversity.

Fractions	Taxonomic *β* diversity				Functional *β* diversity			
	Entire Japanese archipelago including minor islands		Only four main islands		Entire Japanese archipelago including minor islands		Only four main islands	
	*R* ^2^ adjusted observed	*p*‐value	*R* ^2^ adjusted observed	*p*‐value	*R* ^2^ adjusted observed	*p*‐value	*R* ^2^ adjusted observed	*p*‐value
	**Taxonomic *β***_**total**_				**Functional *β***_**total**_			
*Unique*								
S|(E∩H)	**.110**	.010	.029	.146	**.084**	.027	.053	.106
E|(S∩H)	.047	.078	**.074**	.035	**.073**	.018	**.085**	.029
H|(S∩E)	.029	.169	.023	.215	.043	.085	.042	.124
S∩E|H	.039	.065	.028	.077	**.045**	.039	.001	.494
E∩H|S	.032	.076	.005	.435	.020	.204	−.016	.714
S∩H|E	**.100**	<.001	.012	.278	**.059**	.019	−.001	.524
E∩S∩H	**.245**	<.001	**.302**	<.001	**.239**	<.001	**.284**	<.001
*Total*								
Total S	**.494**	<.001	**.371**	<.001	**.427**	<.001	**.336**	<.001
Total E	**.363**	<.001	**.409**	<.001	**.376**	<.001	**.354**	<.001
Total H	**.405**	<.001	**.343**	<.001	**.361**	<.001	**.308**	<.001
	**Taxonomic *β***_**repl**_				**Functional *β***_**repl**_			
*Unique*								
S|(E∩H)	.081	.198	−.046	.782	.060	.215	−.086	.837
E|(S∩H)	.023	.351	.037	.297	.086	.079	.043	.297
H|(S∩E)	.047	.221	−.015	.567	.082	.078	−.012	.550
S∩E|H	−.036	.735	.017	.346	.033	.243	.040	.280
E∩H|S	.023	.331	.031	.294	.028	.261	.008	.454
S∩H|E	**.146**	.013	.019	.330	.045	.209	−.018	.622
E∩S∩H	**.311**	<.001	**.539**	<.001	**.320**	<.001	**.523**	<.001
*Total*								
Total S	**.503**	<.001	**.529**	<.001	**.458**	<.001	**.460**	<.001
Total E	**.322**	<.001	**.624**	<.001	**.467**	<.001	**.614**	<.001
Total H	**.527**	<.001	**.574**	<.001	**.475**	<.001	**.502**	<.001
	**Taxonomic *β***_**rich**_				**Functional *β***_**rich**_			
*Unique*								
S|(E∩H)	.100	.157	.061	.126	.106	.108	**.208**	.015
E|(S∩H)	.052	.215	.099	.082	.048	.217	.131	.064
H|(S∩E)	.006	.407	.046	.201	−.021	.578	**.152**	.030
S∩E|H	**.162**	.002	**.083**	.015	**.096**	.035	−.055	.832
E∩H|S	.027	.299	−.071	.908	.017	.381	−.075	.904
S∩H|E	.051	.227	.016	.345	.063	.146	−.043	.810
E∩S∩H	.023	.341	−.008	.590	.043	.219	−.017	.634
*Total*								
Total S	**.336**	<.001	**.151**	.005	**.308**	<.001	.093	.080
Total E	**.264**	<.001	.103	.051	**.204**	.001	−.016	.553
Total H	**.107**	.021	−.016	.575	**.101**	.031	.017	.314

*Note*: The *p*‐values estimated by 9999 permutations and confidence intervals were set at 95%. The significant factors are bolded. S, E, and H indicate spatial, environmental, and historical factors, respectively.

## 4 DISCUSSION

### 4.1 Biogeographical borders

Our results support our hypothesis that the Tokara Strait acts as a biogeographical border, which is a historical process in Japanese bat assemblages (Figure 1). The Tokara Strait (i.e., Watase line) is recognized as the border between the Palearctic and Oriental realms (Motokawa & Kajihara, 2017). Watase line has been identified as demarcation line affecting species composition in various taxa, including seed plants (Nakamura et al., 2009), land birds (Yamasaki, 2017), non‐volant mammals (Millien‐Parra & Jaeger, 1999), amphibians, and reptiles (Ota, 2000). NNS, south of Tokara strait, was connected to the southern part of the Asian continent; this land‐connection history differs from that of the other regions, and NNS has been isolated for longer than other regions in the Japanese archipelago (Motokawa & Kajihara, 2017). In addition, the deep submarine canyons forming sea barriers, which continue to divide under marine regression during glacial periods, appear to impose constraints on bat dispersal, analogous to their effects on the taxa mentioned above. The dissimilarity between assemblages may reflect geohistorical disparities.

Contrastingly, our results suggest that the Tsugaru Strait does not function as a decisive boundary for bat assemblage composition, with a minor border identified in central‐western Hokkaido rather than within the strait (Figure 2). While the Tsugaru Strait may limit the dispersal of certain species (e.g., *Pipistrellus endoi*), 17 species, almost half of the species in this study, are shared between the HKD and HSK regions (Table S1). The minor border identified in our study aligns closely with the Ishikari lowlands, which is a boundary for several types of insects, called the Kono line (Kono, 1933). This region marks transitional boundaries for the aquatic‐perennial plant species *Nymphaea pygmaea* and *N. tetragona* (Naito & Shiga, 2023). In terms of animals, the border of *Ursus arctos* and *Hynobius retardatus* lineages is located in this lowland (Azuma et al., 2013; Matsuhashi et al., 1999). In terms of bats, elevation difference is indicated to determine community composition (Carvalho et al., 2023; Cisneros et al., 2014). Umbrello et al. (2022) reported that the lowlands act as a dispersal barrier for a cave bat species due to lack of suitable roost locations. Therefore, lowland areas may act as barriers to the dispersal of mountainous or cool‐temperature‐tolerant species due to unsuitable elevation and lack of caves. Based on a combination of the processes with a longitudinal climate gradient, this lowland barrier appears to impose stronger dispersal limitations on bats than the strait, such as the distribution of *Myotis petax*, which inhabit only central and eastern HKD in this study area, representing a more straightforward spatial and environmental barrier. Notably, Kuchinoerabu Island exhibited a distinct assemblage composition within the HSK region (Figure S3). This difference is attributed to *Pteropus dasymallus* and *Tadarida latouchei*, which inhabit NNS and were only found on Kuchinoerabu Island within HSK in our study islands (Table S1, Figure S6).

The contrast in the effectiveness of the two straits as barriers to dispersal could be influenced by several factors. Firstly, past climate change has a notable impact on the persistence of various species (Sandel et al., 2011; Svenning et al., 2015). In the case of bats, tropical niche conservatism related to past climate change affects their current diversity pattern (Stevens, 2006, 2011). Barreto et al. (2021) examined global insular mammal diversity and revealed that islands with lower temperatures tend to have fewer bat species, indicating that specific lineages may struggle to survive in such environments. The NNS region in south HSK is subtropical and likely experienced a more favorable climate under past global cooling than the northern region. In Japanese bats, we observed a decrease in the number of families with increasing latitude and a higher proportion of Vespertilionidae species in our assemblage data (Table S1). These findings suggest that many lineages have been conserved due to milder climates during past climate changes, including global cooling (Barreto et al., 2021; Svenning et al., 2015). This historical process may have prevented mainland species from colonizing the islands by priority effects from species already established on the islands, promoting endemism. Secondly, the varying roles of the two straits as dispersal barriers may be influenced by the distances between regions across the sea. The distance between islands may be a critical factor affecting the success of dispersal. Additionally, the Tokara Strait is characterized by greater sea depths (>200 m; Osozawa et al., 2012) compared with the Tsugaru Strait (130 m; McKay, 2012), resulting in differences in the degree of separation between pairs of regions under sea regression. Consequently, the likelihood of colonization and dispersal may determine whether a strait functions as a biogeographical border.

### 4.2 Factors determining the composition of assemblages

Variation partitioning showed that the fraction shared by all three factors explained the largest portion of *β*_total_ and *β*_repl_ in taxonomic and functional diversity at both scales (Table 1). More than 20% of *β*_total_ and *β*_repl_ at both scales was explained by the shared fraction and the values are higher than those reported in previous studies with similar frameworks (e.g., 2% for Neotropical bats: Varzinczak et al., 2018; 14% for primates: Gavilanez & Stevens, 2013), though we used a different index in our study. This result indicates high collinearity among these factors in the Japanese Archipelago. The long, narrow shape from north to south of the archipelago might form a latitudinal gradient of environments without longitudinal gradients; thus, environmental variables vary more simply with spatial distance than continents with longitudinal gradients. The sample units on a large scale (approximately 6400 km^2^) also affected the pattern because the particular topology or climate was more standardized on a larger scale than a smaller scale. Variation partitioning successfully identified the relative contribution shared among the factors and the contribution of the independent factors and found several significant effects.

On the entire‐archipelago scale, pure spatial processes significantly influenced *β*_total_ at both scales (Table 1). This result corresponds to that of Gavilanez and Stevens (2013), who examined the influence of these three processes on the community composition of Neotropical primates and found that spatial factors explained the variation in beta diversity more strongly than the other factors. In addition, the strong influence of Tokara Strait representing past land connections was indicated by the significant influence of the shared fraction of spatial and historical processes on taxonomic and functional *β*_total_, supporting the results of the cluster analysis. The strait is located in the southern part of the archipelago and borders the assemblages to the north and south. These variations can be explained by geographical distance and differences in geological history; thus, the effect of the strait might be assigned to a shared fraction of the historical process with the spatial process. In addition, the shared fraction between spatial and historical processes significantly influences taxonomic *β*_repl_. This result suggested that the species composition was determined by historical processes, differentiating the species source and causing speciation. Previous studies that examined the historical processes shaping the diversity patterns of bats suggested that the origins of lineages and their dispersal history play an important role in the current patterns of bat diversity (Arita et al., 2014; Stevens, 2006), and support our result. However, functional *β*_repl_ was not significantly determined by the influence of purely spatial processes nor shared by spatial and historical processes. Alternatively, the purely niche‐based processes significantly determined functional *β*_total_ at both scales and the fraction shared among all three processes significantly determined functional *β*_total_ and *β*_repl_. These results suggest that the consistent environmental filter along the archipelago determines the trait composition of bat assemblages rather than the historical processes, in contrast to species composition.

Contrary to these results, at the main islands scale, purely spatial processes and shared spatial and historical processes did not have a significant influence. The purely niche‐based processes significantly explained the taxonomic *β*_total_, which suggests that species composition, including turnover and richness, is determined by niche‐based processes rather than spatial and historical processes at this scale. This observation aligns with our initial hypothesis, suggesting that niche‐based processes primarily drive bat assemblages. Bats are more strongly influenced by environmental factors than other mammals due to their exceptional dispersal capabilities (Monadjem et al., 2023; Varzinczak et al., 2018). Dobrovolski et al. (2012) suggested that a greater disperser can track spatiotemporal environmental dynamics. Consequently, current environmental factors, independent of spatial influences, tend to correlate strongly with these highly mobile species. Bats possess functional traits particularly responsive to environmental gradients, such as climate (Conenna et al., 2021) and urbanization (Jung & Threlfall, 2018). For instance, Conenna et al. (2021) conducted a global examination of bat functional traits in relation to aridity. They found that bats inhabiting arid regions tend to have larger bodies with narrow, elongated wings. This adaptation is believed to respond to the challenges posed by patchy and ephemeral resources, such as water, necessitating greater mobility. This evidence underscores that environmental filtering shapes bat diversity patterns through adaptation to each habitat. It is important to note that our analysis of the main islands scale was limited to the assemblages of the four main islands and did not encompass the islands located south of the Tokara Strait. This absence of clear biogeographical borders in the analysis may have influenced these results. The difference in the results between the two scales indicates that the primary process driving the composition depends on the quality of the dispersal barriers between the terrestrial and aquatic barriers for bats. Sea barriers strongly prevent bat dispersal, and it has been reported that whether a bat is limited to moving islands depends on its vagility and ecology, such as tolerance to open habitats (Heaney et al., 2005). Presley and Willig (2008) examined the factors determining the species composition of bat assemblages in the Caribbean islands and found that inter‐island distance had a greater effect than other factors (e.g., island area and maximum elevation). This result can be interpreted as the composition of assemblages on the islands being determined by the spatial factors of the sea. At the entire‐archipelago scale, islands belonging to the NNS region have both distinct and shared species from other regions. The number of shared species gradually decreased with increasing distance from the HSK region (Table S1), possibly because of the decreasing probability of dispersal and colonization by HSK. Similarly, it has been reported that the geographical pattern of butterfly species composition in the NNS region is strongly controlled by dispersal limitations from the source regions (Hirao et al., 2015). This mechanism may have affected our results. Regarding dispersal limitations, Munguía et al. (2008) examined the area cover of the actual distribution range to the potential distribution predicted by ecological niche modeling in Mexican mammals. They indicated higher occupancy in the potential distribution of bats compared with non‐volant mammals. The authors discussed that a higher dispersal ability makes them less sensitive to geographical barriers and consequently affects their results. In our study, although several pairs of mammals, such as *Craseomys smithii* and *C. andersoni*, were distributed parapatrically (Ohdachi et al., 2015) by paleoclimatic events and geological barriers (Honda et al., 2019) in the HSK region, bats did not show such a distribution pattern. This suggests that Japanese bats are less sensitive to the dispersal limitations of terrestrial geographical barriers. Therefore, high dispersal ability might enable bats to overcome the dispersal limitation of geographical topology and trace the spatiotemporal dynamics of environments; consequently, the niche‐based processes predominantly drive the pattern of species composition of bats at the main islands scale.

The fraction shared by spatial and niche‐based processes significantly determined taxonomic and functional *β*_rich_ at the entire‐archipelago scale. In this study, niche‐based processes included island size, and it is considered that the fraction of island size was shared with spatial factors because the minor islands were particularly isolated. Thus, the result of taxonomic *β*_rich_ indicates that the species‐area relationship (SAR) on islands determines the differences in species richness among assemblages, which is consistent with bat assemblages in other regions (Carvajal & Adler, 2005; Carvalho et al., 2021; Frick et al., 2008; Ricklefs & Lovette, 1999). Our results are supported by reports of SAR in non‐vorant mammals in the Japanese Archipelago (Millien‐Parra & Jaeger, 1999). The results of functional *β*_rich_ also suggest that functional diversity correlates with island size. This is consistent with previous studies about the relationship between functional diversity and island size (Jacquet et al., 2017; Si et al., 2017; Whittaker et al., 2014). This result was considered to be influenced by both variance of species richness (Swenson, 2014) and pure variation of island size (Jacquet et al., 2017; Ross et al., 2019). In the future, partitioning between the influences of species richness and island size on functional diversity to understand the primary process of community assembly in Japanese bats in detail.

## 5 CONCLUSIONS

Our hypotheses have garnered support, revealing that the historical‐spatial processes, representing past land connections, and niche‐based processes influence the current taxonomic and functional diversity of bats. Notably, the effects of these processes are contingent on the specific scale of focus. Shared historical and spatial processes predominantly drive the composition of bat assemblages in the entire archipelago, including small minor islands. However, a niche‐based process emerges as the primary determinant of taxonomic diversity when narrowing the focus to the larger main islands. This contrast with previous studies conducted in continental zones (Batista et al., 2020; Monadjem et al., 2023; Varzinczak et al., 2019) and island regions (Presley & Willig, 2008) underscores the heightened impact of historical processes on bat species composition within regions comprised by different geohistorical islands. On the other hand, our study found that niche‐based process explains a greater fraction of functional diversity than of taxonomic diversity. This finding shows that species composition is determined by the historical factors of source difference and speciation, while the functional composition is determined by consistent environmental gradient. Moreover, the divergent outcomes observed at different scales underscore the intricate relationship between the unique environments of surrounding seas and islands and their influence on bat diversity patterns through distinct processes.

Our study represents the first comprehensive biogeographical investigation of Japanese bat assemblages on the underlying determinants of their composition. Notably, we have identified that niche‐based processes, alongside the shared fraction of historical and spatial processes, play a pivotal role in shaping the present bat diversity in the Japanese Archipelago. This information is invaluable in understanding how Japanese bats on the main islands may be highly responsive to environmental changes, including potential future climate shifts and land‐use conversions. Furthermore, our study highlights the significance of the shared fraction between spatial and historical processes, a factor correlated with the high degree of endemism observed in the Nansei Islands (NNS). In Japan, bat research has exhibited notable biases, with less attention directed toward endangered species, particularly those endemics to the NNS (Preble et al., 2021). Our result underscores the pressing need for further investigations to identify species at heightened risk under future environmental changes, thereby facilitating the conservation of endemism in Japanese bat diversity.

## AUTHOR CONTRIBUTIONS

**Takahiro Maki:** Conceptualization (equal); data curation (lead); formal analysis (lead); validation (equal); writing – original draft (lead); writing – review and editing (equal). **Nozomi Sannomiya:** Data curation (equal); writing – review and editing (supporting). **Toshihide Hirao:** Conceptualization (equal); funding acquisition (equal); writing – review and editing (equal). **Dai Fukui:** Conceptualization (equal); funding acquisition (lead); project administration (lead); supervision (lead); validation (equal); writing – review and editing (equal).

## CONFLICT OF INTEREST STATEMENT

The authors have no competing interests.

## Supporting information


Figures S1–S7.

Table S1.

Table S2.

## Data Availability

Distribution data, functional data, R scripts, each of factor‐data are available in a github repository (https://github.com/BatTakaMaki/Japan_Bat_Supplemental).
