# Supplementary material for: Scale‐dependent influences of environmental, historical, and spatial processes on taxonomic and functional beta diversity of Japanese bat assemblages
Source: Ecol Evol. 2024 Apr 15;14(4):e11277. doi: 10.1002/ece3.11277 (PMC11019122; doi:10.1002/ece3.11277)
Supplement: Supplementary file 1 — Figures S1–S7. [file ECE3-14-e11277-s003.docx]

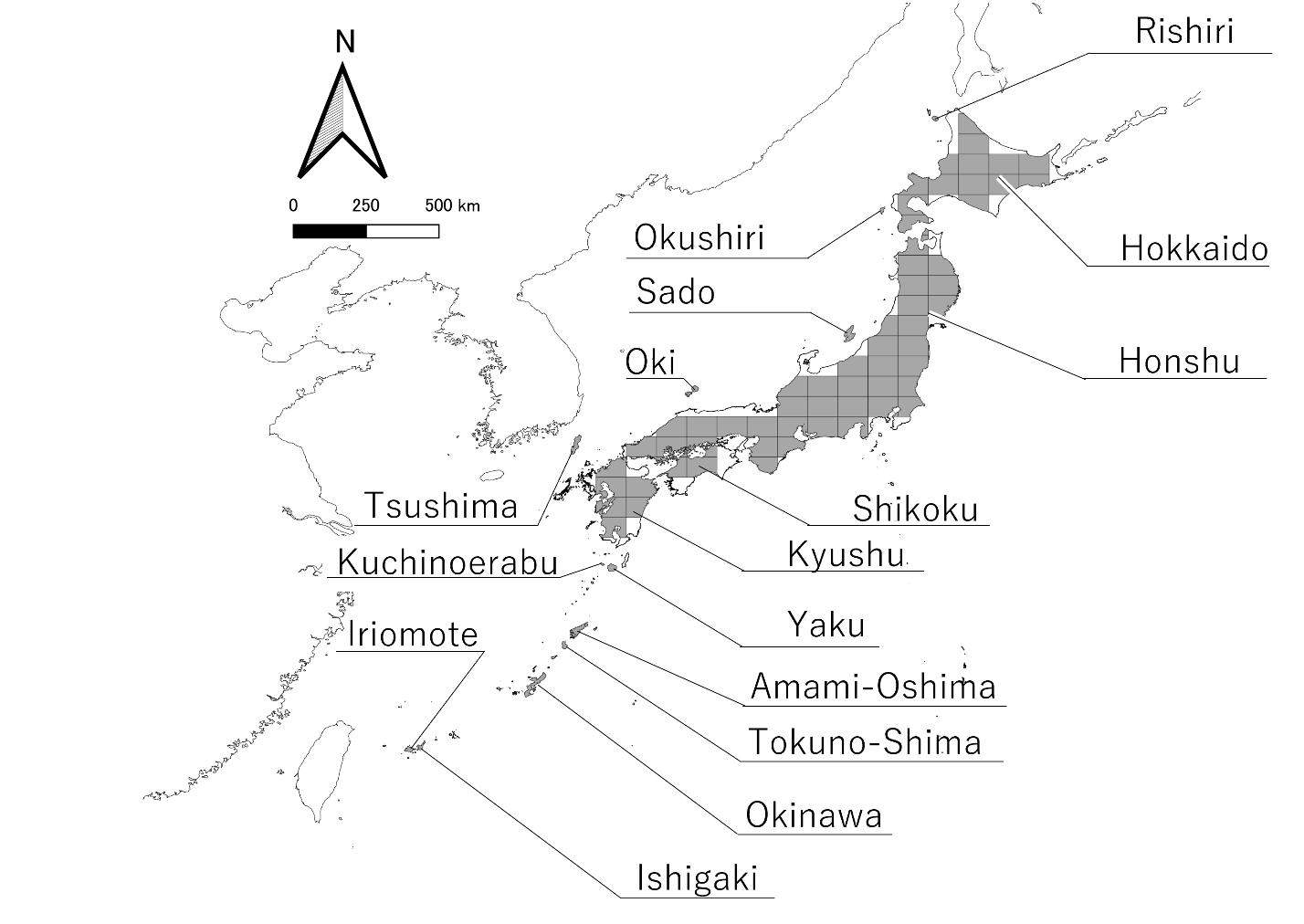


Figure S1. Islands for investigating the composition of bat assemblages in the Japanese Archipelago.


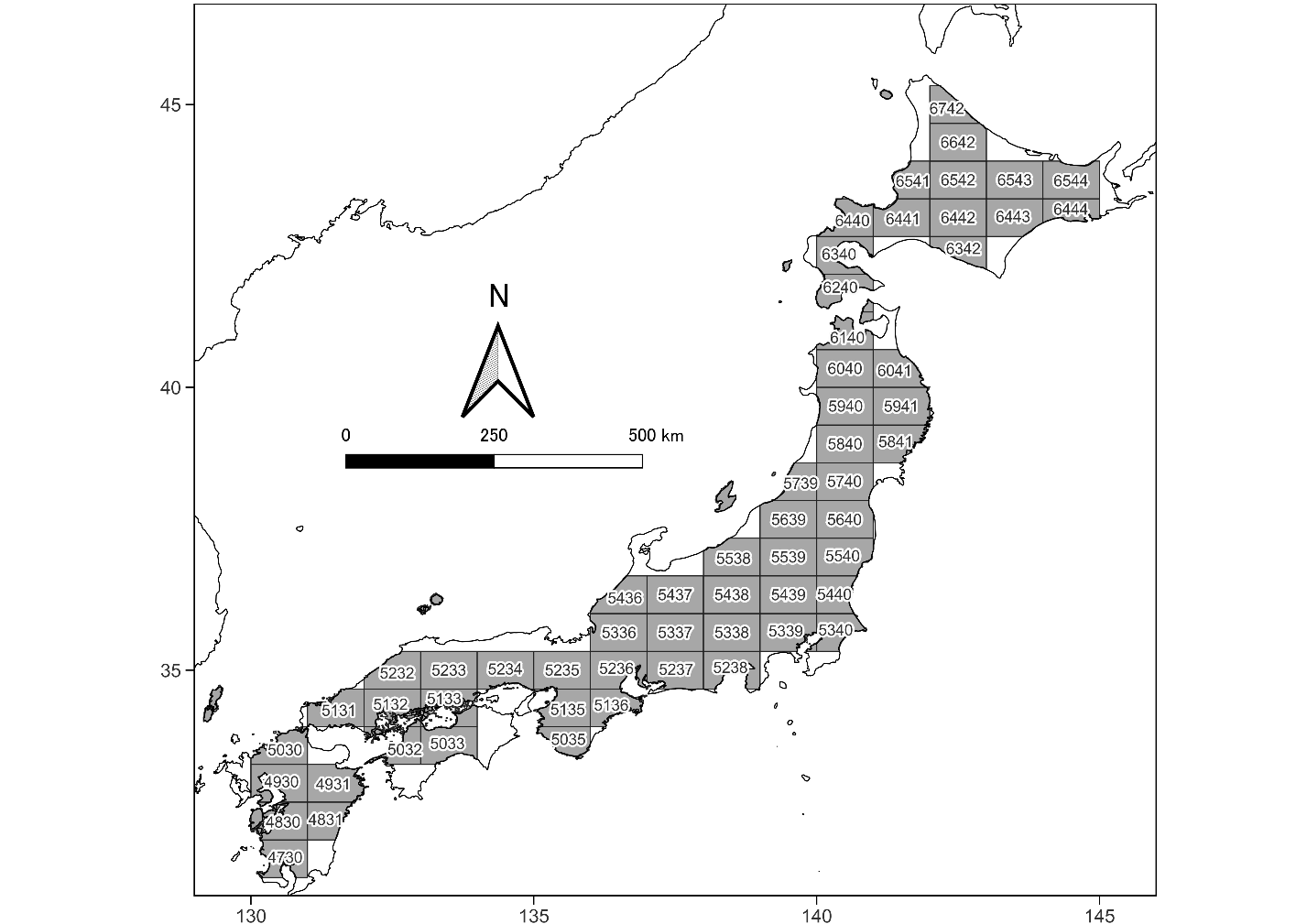


Figure S2. Japan's First Standard Grids with identification number for investigating of bat assemblages in the Japanese Archipelago.


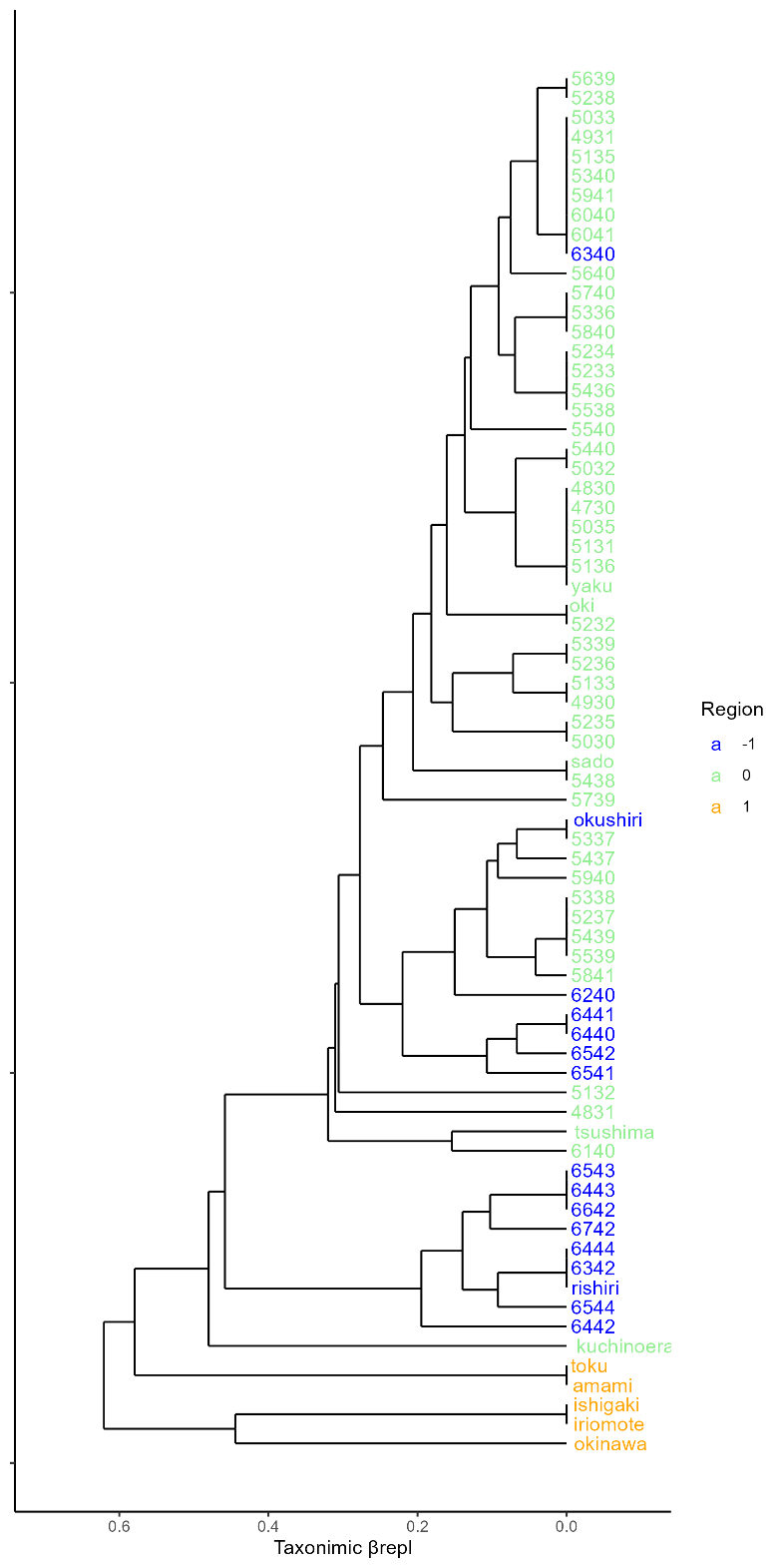


Figure S3. Result of cluster analysis based on taxonomic turnover (β_repl_) in the dendrogram. The name of blue, light green, orange indicate belonging to Hokkaido (HKD) region, Honshu-Shikoku-Kyushu (HSK) region, and Nansei Islands region (NNS), respectively.


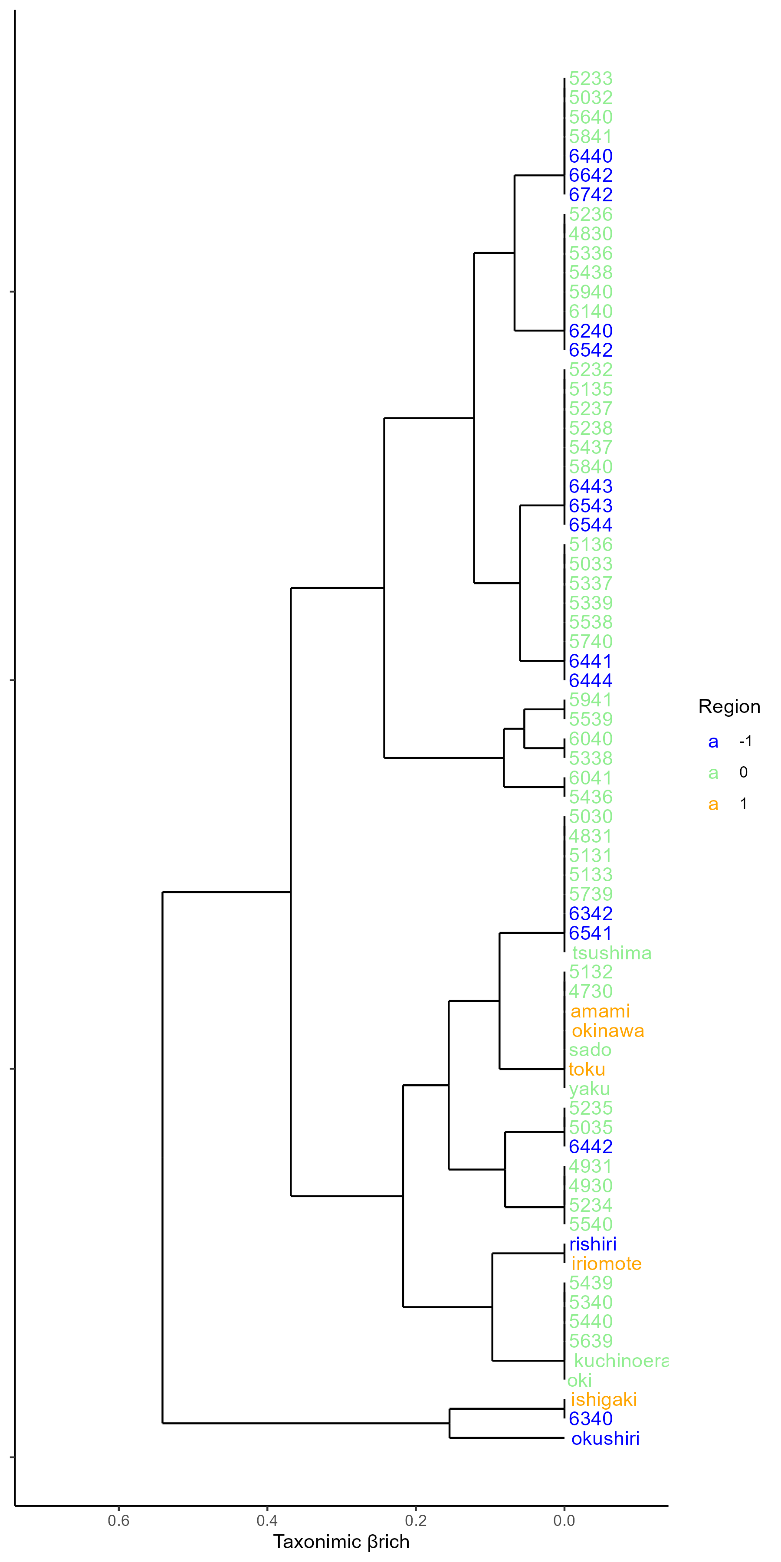


Figure S4. Result of cluster analysis based on taxonomic richness difference (β_rich_) in the dendrogram. The name of blue, light green, orange indicate belonging to Hokkaido (HKD) region, Honshu-Shikoku-Kyushu (HSK) region, and Nansei Islands region (NNS), respectively.


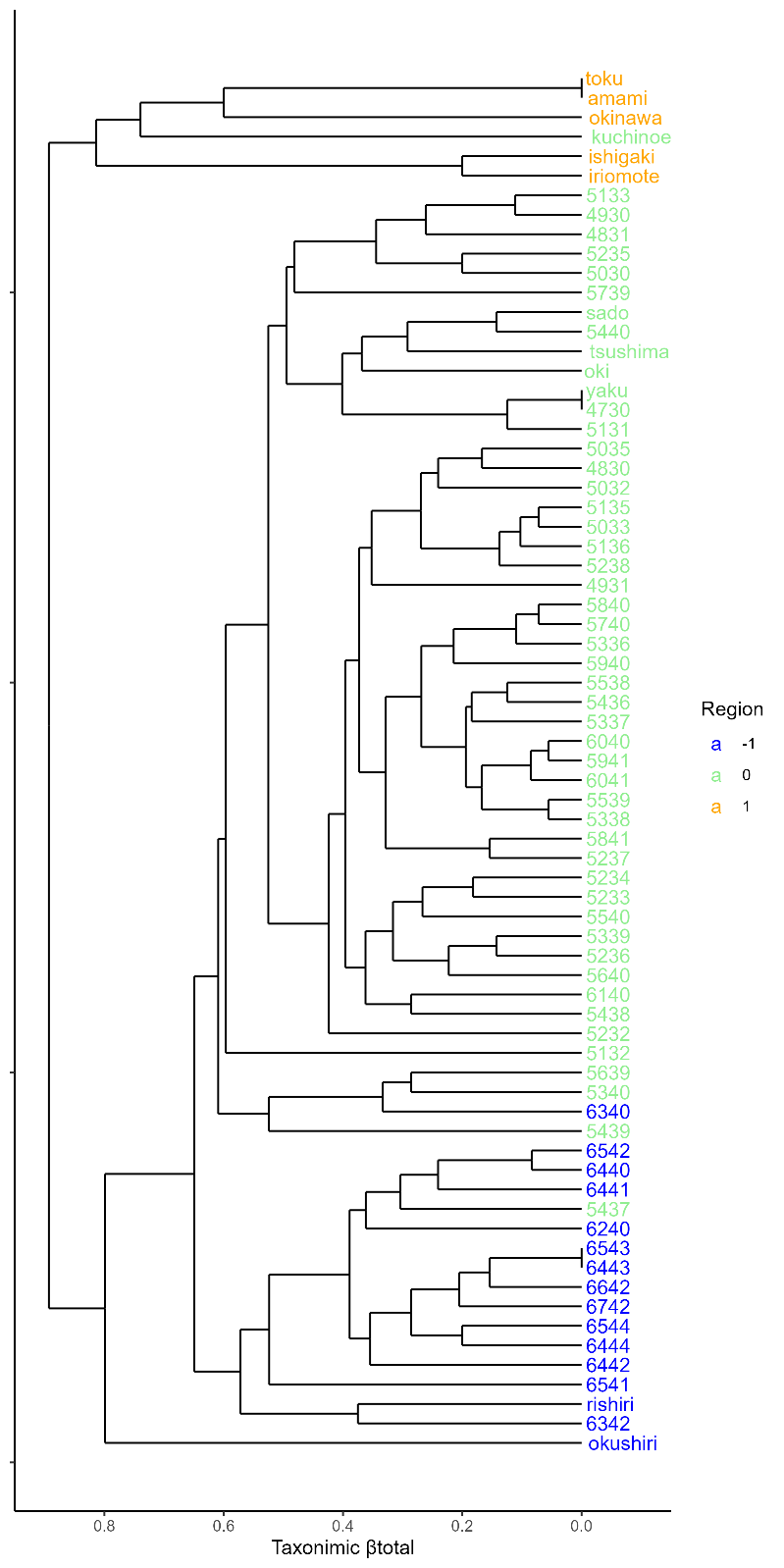


Figure S5. Result of cluster analysis based on taxonomic beta diversity(β_total_) including richness difference and turnover components in the dendrogram. The name of blue, light green, orange indicate belonging to Hokkaido (HKD) region, Honshu-Shikoku-Kyushu (HSK) region, and Nansei Islands region (NNS), respectively.


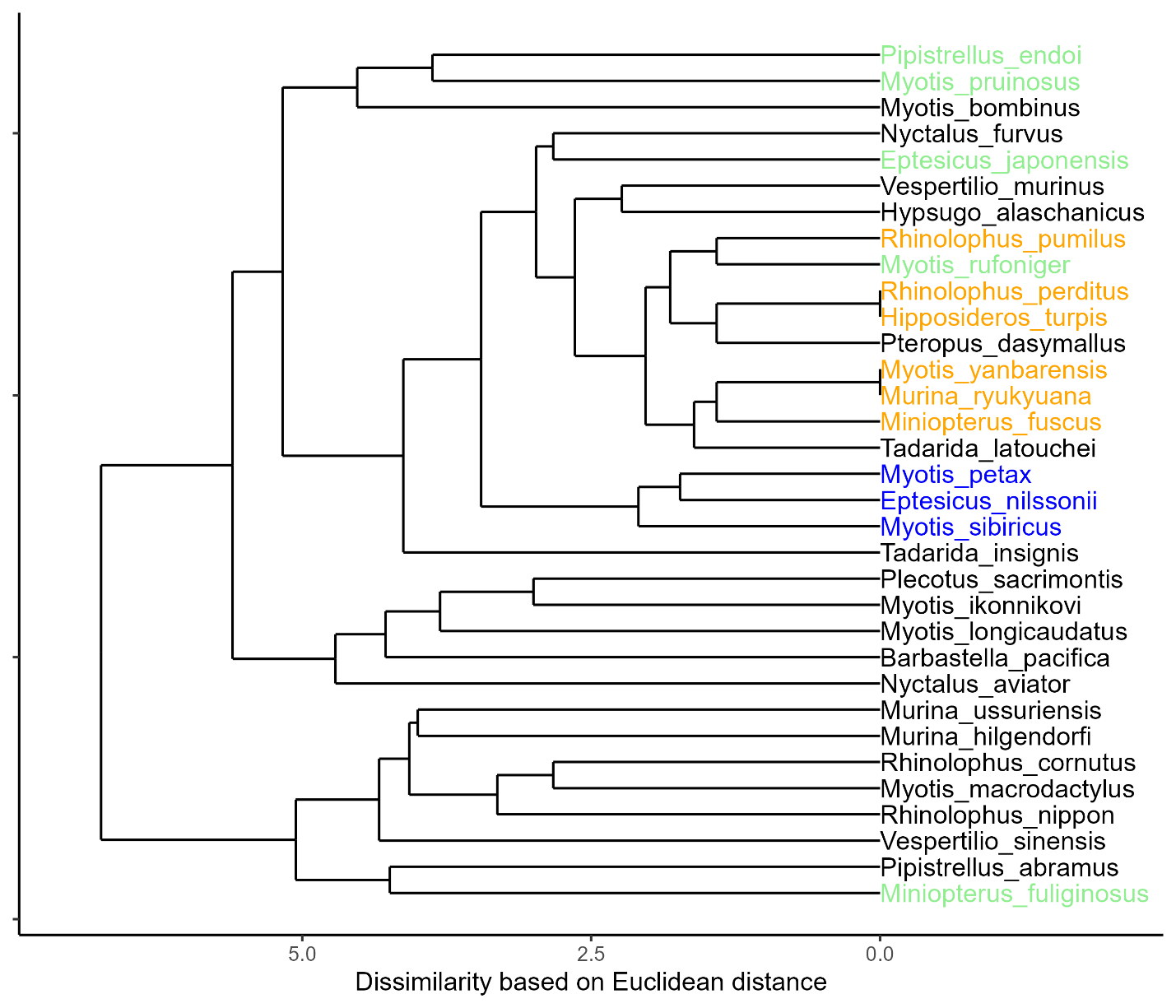


Figure S6. Result of cluster analysis based on species occurrence in assemblages. The name of blue, light green, orange, black indicate only-inhabiting Hokkaido (HKD) region, Honshu-Shikoku-Kyushu (HSK) region, and Nansei Islands region (NNS), and distribution across two or more regions, respectively.


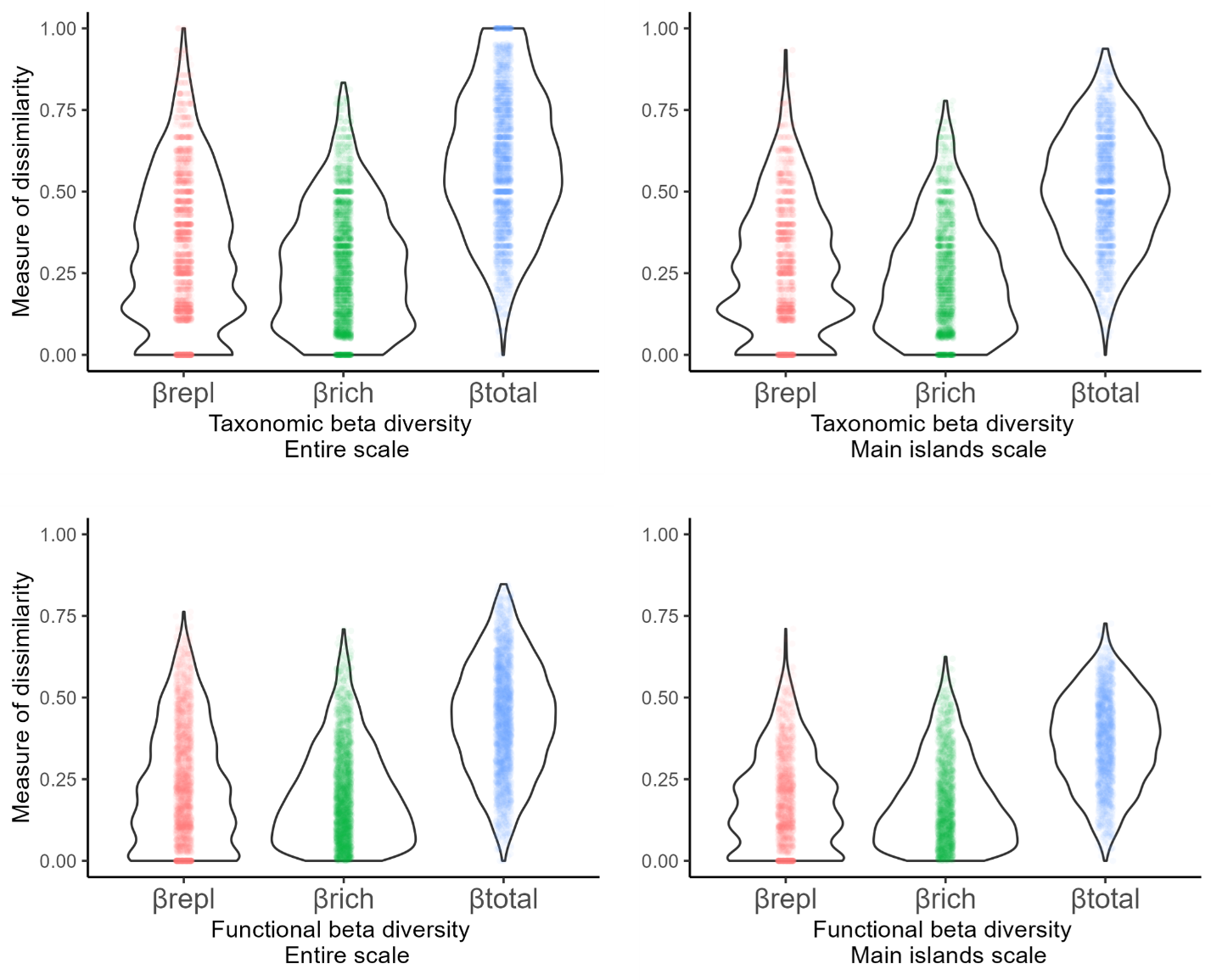


Figure S7. Components of replacement (β_repl_) and richness (β_rich_) and total (β_total_) in pairwise taxonomic and functional beta diversity in each pair of assemblages the Japanese bats.
